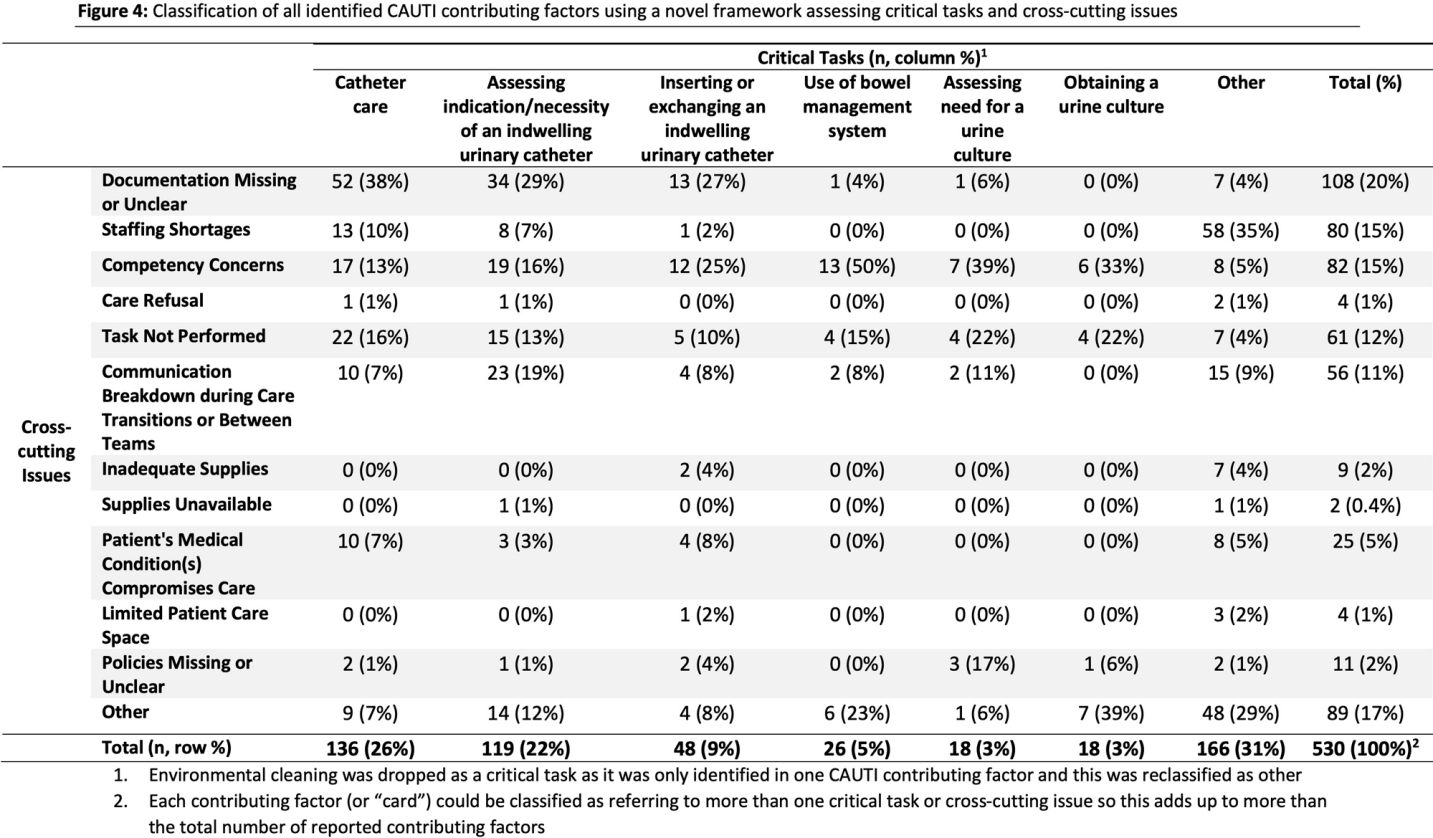# Contributing Factors to Central Line-associated Bloodstream Infections and Catheter-associated Urinary Tract Infections

**DOI:** 10.1017/ash.2024.309

**Published:** 2024-09-16

**Authors:** Jessica Howard-Anderson, Lindsey Gottlieb, Lori Grooms, Carolyn Holder, Lisa Reif, Krystle Johnson, Victoria Dotto, Julianne Kubes, Kari Love, Rachel Regina, David Murphy, Jesse Jacob, Colleen Kraft, Joel Mumma

**Affiliations:** Emory University School of Medicine; Emory University Hospital; Emory Healthcare; Healthcare Human Factors Lab; Emory University

## Abstract

**Background:** Central line-associated bloodstream infections (CLABSI) and catheter-associated urinary tract infections (CAUTI) are key healthcare-associated infection (HAI) quality metrics. In this qualitative analysis, we aimed to identify common issues contributing to CLABSIs and CAUTIs occurring during the COVID-19 pandemic. **Methods:** In an academic healthcare network in Atlanta, GA, four hospitals perform real-time, apparent cause analyses (ACAs) for all CLABSIs and CAUTIs. Contributing factors are entered as free text into an electronic database. We analyzed data from 8/2020–8/2022. We first performed a qualitative open card sort of all reported contributing factors to CLABSI and created a novel framework based on mutually defined critical tasks (e.g., line insertion) and cross-cutting issues (e.g., communication breakdown). Contributing factors could describe ≥1 critical task and/or ≥1 cross-cutting issue. After establishing interrater reliability, a multidisciplinary group applied this framework to classify each contributing factor. For CAUTI, we used the same set of cross-cutting issues but identified new critical tasks via open card sorting. We then used the framework to classify each CAUTI contributing factor. We used descriptive statistics to identify frequent critical tasks and cross-cutting issues. **Results:** We reviewed 350 CLABSI ACAs with 602 contributing factors and 240 CAUTI ACAs with 405 contributing factors (Figure 1). Our classification framework comprised 11 cross-cutting issues and 9 critical tasks for CLABSI and 7 critical tasks for CAUTI (Figure 2). CLABSI: The critical tasks most often reported were bathing (19%), central line dressing maintenance (15%), and assessing central line indication (8%; Figure 3). Within these tasks, the most frequent issues described for bathing were the task not being performed (20%) and unclear documentation (18%); for dressing maintenance, the task was not performed (15%), not documented (15%), or poorly performed due to lack of competency (15%); and for assessing line indication, there was frequent communication breakdown (33%). CAUTI: The critical tasks most often reported were urinary catheter care (26%) and assessing the indication for urinary catheter (22%; Figure 4). Within these tasks, urinary catheter care was frequently not documented (38%) or not performed (16%); assessing urinary catheter necessity was often not documented (29%) or involved breakdown of communication (19%). **Conclusion:** We created a novel framework to evaluate common causes of HAIs in an academic healthcare network. This framework can be used to identify and track gaps over time and to develop quality improvement initiatives targeting key tasks and associated factors, such as communication difficulties when assessing device indications.

**Disclosure:** Colleen Kraft: Consultant - REbiotix/Ferring; Scientific Advisory Board - Seres, LLC

**Figure:**
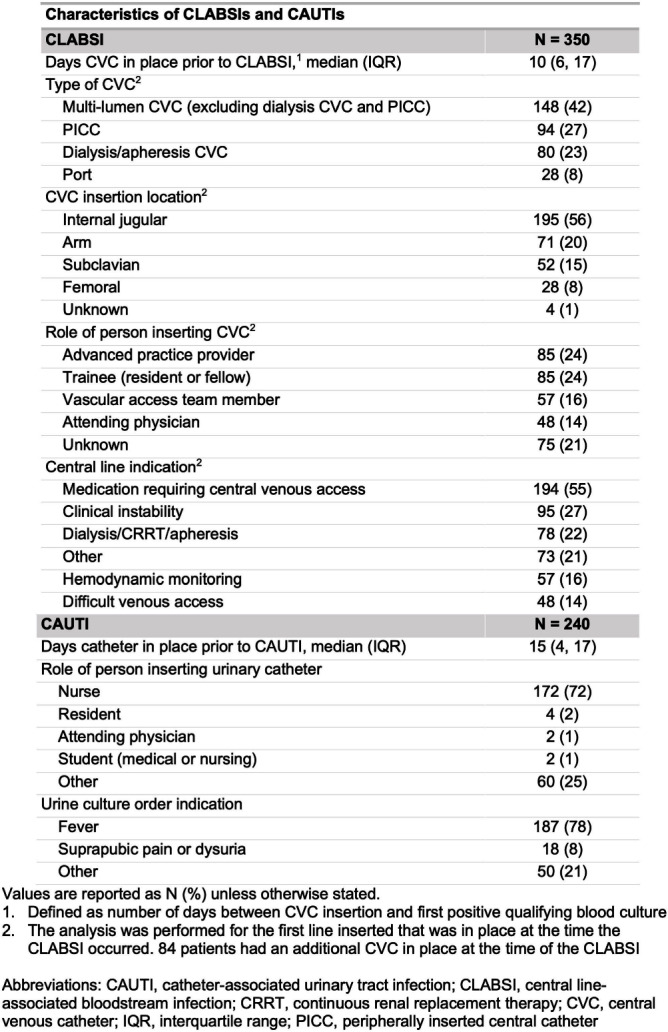

**Figure:**
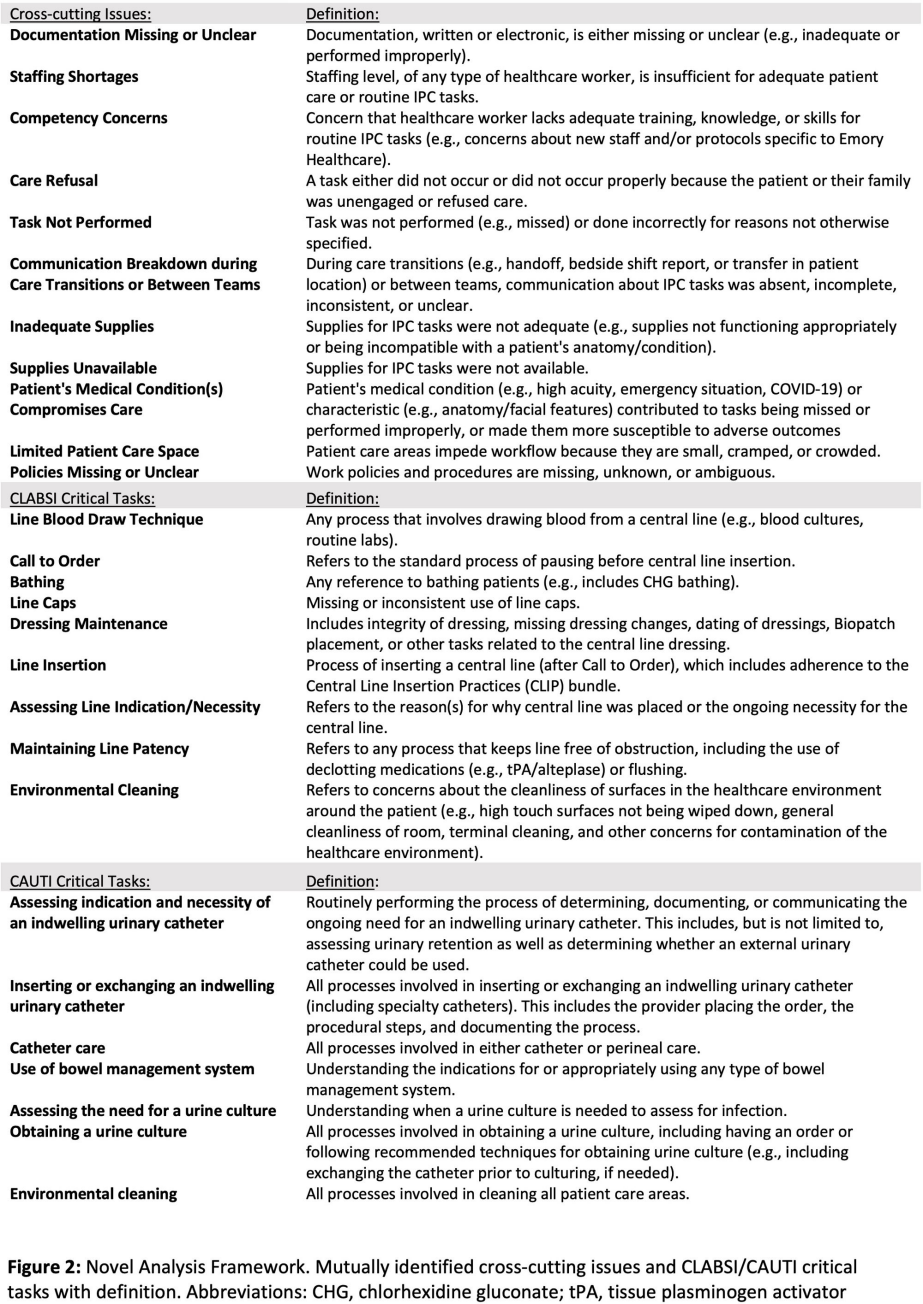

**Figure:**
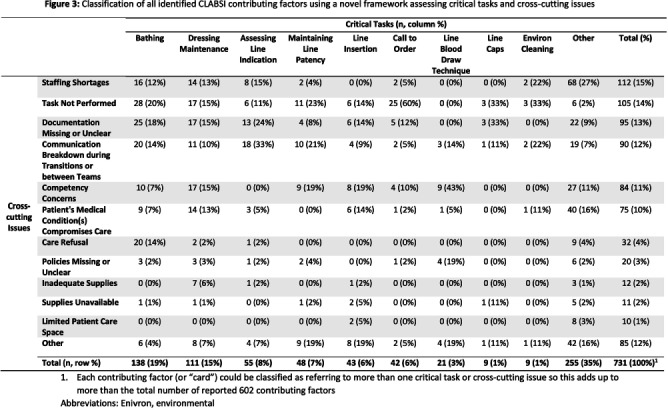

**Figure:**